# Making Healthy, Sustainable Diets Accessible and Achievable: A New Framework for Assessing the Nutrition, Environmental, and Equity Impacts of Packaged Foods

**DOI:** 10.1093/cdn/nzac136

**Published:** 2022-08-27

**Authors:** David I Gustafson, Eric A Decker, Adam Drewnowski, Michael W Hamm, Jane Hwang, Kathleen A Merrigan

**Affiliations:** Adjunct Research Faculty, Biological Systems Engineering, Washington State University, Pullman, WA, USA; Department of Food Science, University of Massachusetts, Amherst, MA, USA; Center for Public Health Nutrition, University of Washington, Seattle, WA; USA; Department of Community Sustainability, Michigan State University, Lansing, MI, USA; Social Accountability International, New York, NY, USA; Swette Center for Sustainable Food Systems, Arizona State University, Tempe, AZ, USA

**Keywords:** sustainability, ultra-processed foods, dietary guidance, NOVA classification, sustainable nutrition

## Abstract

There is a growing global consensus among food system experts that diets and how we source our foods must change. The sustainable nutrition community continues exploring the environmental impact and dietary value of foods. Packaged foods have been largely ignored within the dialogue, and if they are addressed, existing frameworks tend to label them all as “ultraprocessed” and uniformly discourage their consumption. This approach lacks the nuance needed to holistically evaluate packaged foods within recommended dietary patterns. Additionally, there is considerable diversity of opinion within the literature on these topics, especially on how best to improve nutrition security in populations most at risk of diet-related chronic disease. In support of addressing these challenges, 8 sustainability and nutrition experts were convened by Clif Bar & Company for a facilitated discussion on the urgent need to drive adoption of healthy, sustainable diets; the crucial role that certain packaged foods can play in helping make such diets achievable and accessible; and the need for actionable guidance around how to recommend and choose packaged foods that consider human, societal, and planetary health. This article summarizes the meeting discussion, which informed the development of a proposed framework based on guiding principles for defining sustainable, nutritious packaged foods across key nutrition, environmental, economic, and sociocultural well-being indicators. Although additional research is needed to substantiate specific metrics in order to operationalize the framework, it is intended to be a foundation from which to build and refine as science and measurement capabilities advance, and an important step toward broader adoption of healthy, sustainable diets.

## Introduction

The joint crises related to public health, food insecurity, and climate change are driving urgency toward a more sustainable food system that keeps people well, enhances food access, and minimizes environmental impacts to feed generations to come, without depleting natural resources. There are efforts to establish guiding principles on the part of FAO/WHO (1) to define healthy, sustainable diets, and others have tried to integrate such ideas into food policy and dietary guidance. Chief among these other efforts are EAT Lancet (2–5), the 2021 UN Food Systems Summit (6), and the EU Farm to Fork multiscale approach (7). However, existing sustainable nutrition frameworks and proposed diets focus almost exclusively on whole foods. If packaged foods are discussed, they are often uniformly discouraged as “ultraprocessed foods (UPFs)” (8–24), which contributes to making current recommendations challenging to achieve.

Although most packaged foods would be considered UPFs within the widely cited NOVA system (8), the precise definition of UPFs within the broader scientific literature is still quite unsettled, and UPFs vary considerably in their impacts on human, environmental, and societal health (9–11). Although whole foods are the foundation of healthy eating patterns, the facts of modern life and socioeconomic realities necessitate quick and portable options that are also nutritious, equitably available, of high quality, and sustainably produced. The broad set of packaged foods categorized as UPFs—for example, everything from cookies and candy to flavored yogurts, ready-to-eat meals, and whole grain cereals and bars—are a large contributor to global dietary intake, comprising 57% of US diets (12). Given their prevalence, it is essential to identify and define the key characteristics of packaged foods, specifically those classified as UPFs within the NOVA system, that align with healthy, sustainable dietary recommendations to meet people where they are and to ensure these diets are realistic, scalable, and accessible to all.

**FIGURE 1:**
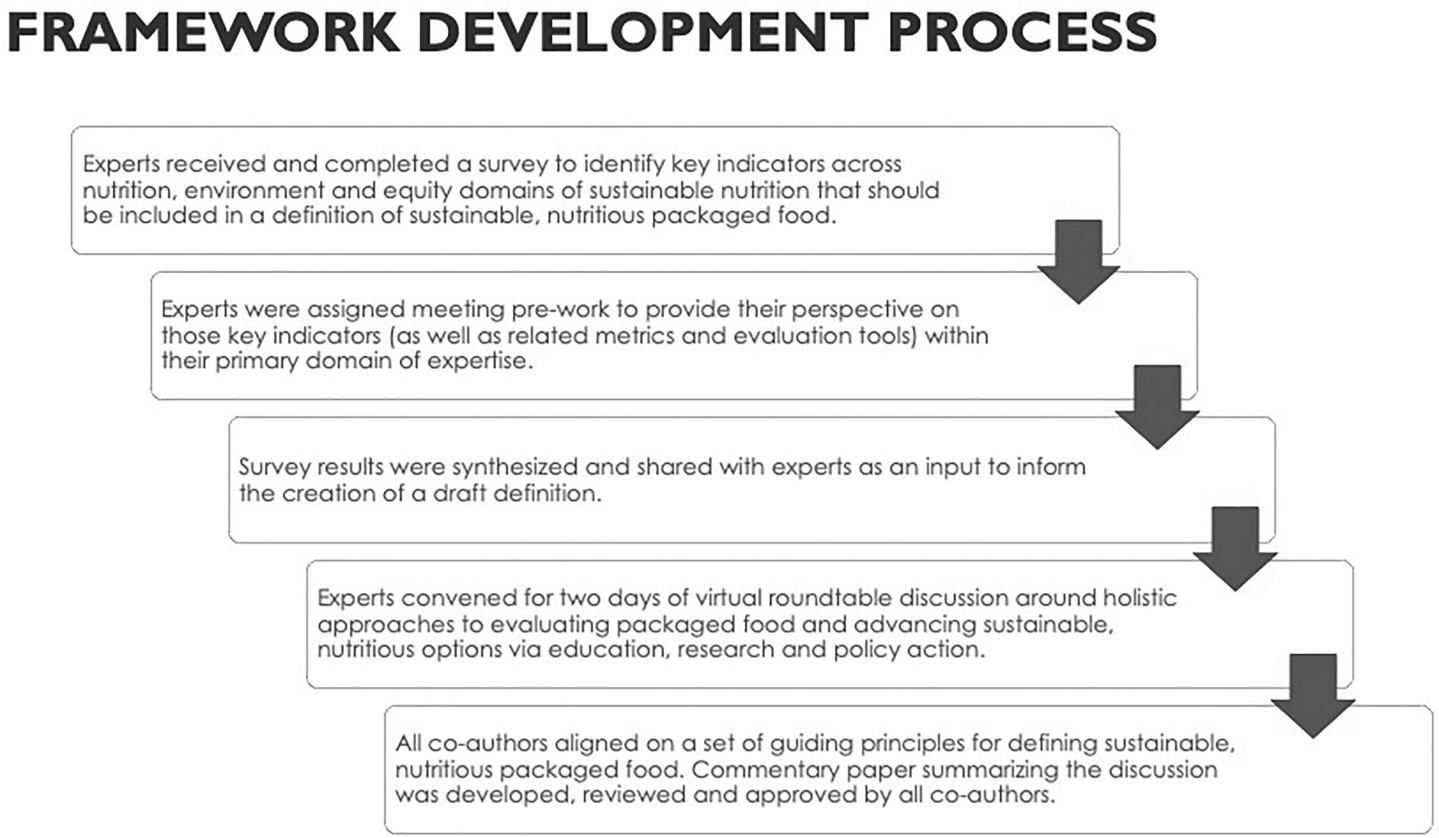
Schematic showing the framework development process.

In support of this goal, Clif Bar & Company convened a group of 8 sustainability and nutrition experts for a facilitated discussion in November 2021 (see **Figure 1** for a schematic of the overall framework development process). Much of the dialogue focused on determining a set of guiding principles for defining sustainable, nutritious packaged foods (SNPFs) and applying them to snacks. Opportunities to advance sustainable nutrition via education, research, and policy action were also discussed. In addition to the 6 coauthors of this article, we solicited input from external experts, including 2 experts who participated in the workshop, to provide background information on the policy and communications landscape to inform the discussion but their contributions were not directly relevant to the development of the framework itself. Accordingly, the collective use of the term “we” in this article reflects a consensus among the 6 coauthors.

## The Role of Packaged Foods in Healthy, Sustainable Diets

Current healthy, sustainable diet frameworks tend to disregard or discourage most packaged foods. However, this approach is inconsistent with the realities of modern life. Packaged foods can help improve food safety, reduce unnecessary food waste, and conserve resources (25), and they have long been recognized as a contributor to food and nutrition security (26). Affordable nutrient density cannot be achieved without packaged foods, as demonstrated in a recent study showing that a combination of unprocessed foods and UPFs is required to generate an affordable food pattern, and that UPFs are essential to creating food patterns that are nutritionally adequate (20). Packaged foods also help save time on food preparation, meal planning, and procurement, and thereby make sustainable eating accessible to a variety of skill levels. Although packaging itself can have environmental costs, packaged foods also have environmental benefits that are often not recognized—the efficiencies of food manufacturing and processing at scale make it more environmentally friendly (using less energy, water, generating less waste, etc.) than preparing food at home in many cases (27). Therefore, packaged foods are a useful part of the solution to make healthy, sustainable dietary patterns achievable and accessible to all. Given that packaged foods vary significantly in their nutrition, equity, and environmental impacts, it is important to have more robust, holistic evaluation systems to inform choices that align with recommended diets.

To date, the most widely cited categorization system proposed for packaged foods is NOVA (8). The NOVA system evaluates foods according to the degree of processing, with the highest degree of processing considered to be UPFs. It is estimated that ∼71% of the packaged foods available in the United States—everything from soda, cookies, and candy to ready-to-eat cereals, whole grain breads and pastas, flavored yogurt, and whole grain bars—would be considered UPFs based on the NOVA definitions (28). This broad classification recommends avoiding many convenient, affordable, nutrient-rich foods, which might serve to further worsen health disparities. A recent commentary (24) suggests that the NOVA system could be too blunt to guide public health responses and that uniformly reducing all UPFs could have unintended harms, including detrimental effects on nutrition security. Of particular importance to the current discussion, the NOVA system does not fully address critical aspects of sustainability (17), suggesting a need to build out an alternative evaluation system that also considers environment and social equity considerations. On the environmental aspects of UPFs, the literature is still in its infancy, with 1 recent study showing weak linkages to water footprints, but not to carbon footprints (11).

Whereas numerous epidemiological studies have shown an association between UPF consumption and health risks, including cardiovascular disease (9), weight gain (13) and diabetes (14), reduced diet quality (22), and overall mortality (10), to date there is only 1 randomized, controlled human clinical study (19) and limited understanding of the mechanism(s) of action. In addition, a recent analysis highlighted concerns with the functionality of NOVA in its current form due to notable inconsistencies regarding how foods were categorized, calling into question the reliability of conclusions from previous epidemiological studies (29).

Although the antiprocessing sentiment of NOVA has gained some traction globally, it is certainly not universally accepted. For instance, the current US Dietary Guidelines (30) note that although preparing meals at home can help support healthy habits, it is not realistic or desirable to avoid the purchase and consumption of foods prepared by others given consumers’ limits on time and desire for convenience.

The scientific community is still debating the implications of UPFs given their broad definition and lack of distinction between different types of foods. In fact, nutrient profiling systems appear to have a stronger correlation with healthy eating patterns. A study (16) comparing NOVA with pre-existing indices concludes that the NOVA classification scheme adds little to the pre-existing nutrient profiling models, noting that the purported links between NOVA categories and health outcomes could have been obtained using Nutrient Rich Foods nutrient density metrics. We assert that for truly relevant and applicable dietary guidance, consideration of packaged foods must expand beyond processing into a framework that includes considerations for nutrition, environment, and equity and can be evolved along with science and measurement capabilities.

## Defining SNPFs: A Proposed Framework

Although some other frameworks for evaluating diets and individual food items across the various dimensions of sustainable nutrition have been proposed (13, 31–33), they were not intended for and do not adequately address all aspects of packaged foods. The current ways of evaluating packaged foods are not adequately informing choices that are aligned with healthy, sustainable diet recommendations and there is a pressing need for new definitions given the considerable variation in their impacts on human, environmental, and societal health. Most existing profiling systems address nutrition (e.g., Nutri-Score, Health Stars) or sustainability (e.g., Eco-Score) attributes in isolation, so our intent was to build upon these efforts to develop an integrated framework that offers a more holistic evaluation approach.

**FIGURE 2:**
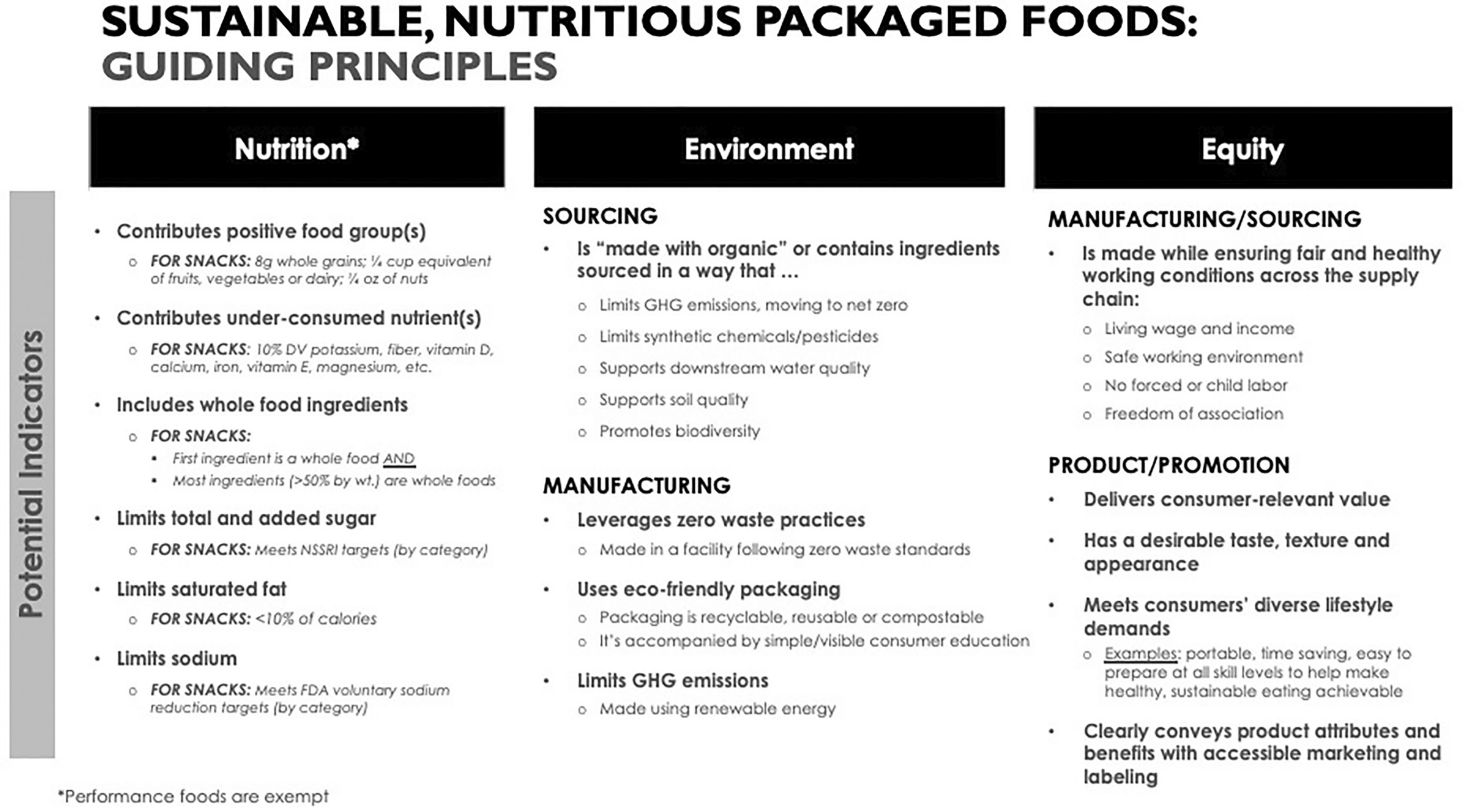
Proposed guiding principles for the definition of sustainable, nutritious packaged foods (SNPFs), including specific nutrition criteria for snacks, exclusive of performance foods. DV, recommended daily intake; GHG, greenhouse gas; NSSRI, National Salt and Sugar Reduction Initiative.

Our discussions resulted in the development of a framework of guiding principles for defining SNPFs in 3 domains: Nutrition, Environment, and Equity, as presented in **Figure 2**. Environmental impacts are considered separately for the sourcing of food ingredients and for manufacturing steps. Equity factors are similarly divided into 2 categories: manufacturing/sourcing and product/promotion. Space does not permit a complete record of the 2 days of deliberations that resulted in the final set of guiding principles, but the highlights are provided here.

Prior to the workshop, each participant completed a survey regarding key indicators or characteristics across nutrition, environment, and equity (encompassing sociocultural and economic considerations) domains of sustainable nutrition that should be included in a definition of SNPFs. The survey responses helped identify areas of consensus, prioritize areas of focus, and inform a draft definition for our deliberation. All coauthors were asked to provide their perspective on key indicators, metrics, and evaluation tools, as well as challenges and limitations when considering application to packaged foods. As the coauthors of this article, we ultimately achieved consensus on a framework of key guiding principles for defining SNPFs. Key points of discussion are summarized below.

### Nutrition

We aligned on the inclusion of positive food groups, positive nutrients, whole food ingredients and limited sugar, saturated fat, and sodium as the key indicators to assess nutrition contributions of packaged foods. The nutrition parameters might need to be adapted by food category as is currently done in many nutrient profiling systems (16), including the National Salt and Sugar Reduction Initiative (34), Choices International (35), and the US Children's Food and Beverage Advertising Initiative (36). For example, a packaged meal could be expected to contribute more positive food groups and nutrients or different limits on sugar, saturated fat, or sodium than a snack. We discussed the appropriateness of processing as an indicator, and agreed that processing was not, in and of itself, a key determinant of the nutrient density or well-being benefits of a food. More important is food formulation, which is a direct indicator for higher fat, sugar, and salt foods. There is so much variety in what is currently considered a UPF that advice to avoid all foods in that category can negatively impact nutrition security.

We agreed that the future of food development should align with the 4 previously proposed domains of improved diet quality (37)—moderation (e.g., the use of new technologies to drive reduction of saturated fat, added sugar, sodium), balance (e.g., the balance of animal compared with plant protein), adequacy (e.g., fortification with nutrients of concern like calcium, vitamin D, folate, fiber), and diversity (e.g., a wide variety of whole food ingredients across the food groups)—and to consider cultural sensitivities and support of regional food-based dietary guidelines in any definition of an SNPF. We discussed the importance of developing foods according to these principles that align with other consumer drivers of food choice (e.g., taste, price, convenience).

As dietary guidance moves toward food-based guidance and away from nutrient-specific guidance, emphasizing whole food contributions via food groups and ingredients in the definition will be essential.

### Environment

Environmental indicators of SNPFs were considered from both the sourcing and manufacturing aspects of production. We agreed that the key ingredient sourcing indicators were to limit greenhouse gas (GHG) emissions, improve soil quality, limit synthetic pesticides, and promote biodiversity. We also agreed upon including impacts on downstream water quality as a key indicator, given the significant benefits that sustainable sourcing can have there.

In terms of how to best quantify and measure GHG emissions, we agreed that the ultimate goal should go beyond limiting emissions and toward net zero, including carbon sequestration in the crop/animal production stage. Getting life cycle assessment data for all products and ingredients would be challenging now due to data scarcity so it might not be currently possible to quantify this precisely by product. Another approach could be to look at dominant ingredients for net carbon footprint, specific to the region where sourced. Although not the only option for assessing the sustainability impacts of foods and ingredients, organic certification is an existing, robust certification that could offer a way to evaluate some prioritized key environmental indicators. Although there are complexities and trade-offs associated with organic agriculture (e.g., lower yields, additional costs), it was advanced as the primary environmental sourcing consideration given the current lack of consistent data for the other recommended indicators.

When considering manufacturing characteristics of SNPFs, we were aligned on the pursuit of zero waste to the maximum feasible extent, eco-friendly packaging, and producing food in manufacturing facilities that limit GHG and other emissions that harm people in surrounding communities. Related to packaging, the importance of consumer education to clearly communicate how the products can be recycled, composted, etc. was also raised. Packaged foods manufactured at scale can come with sustainability and affordability benefits over items prepared at home or in smaller operations—for example, buying raw materials in large quantities, using energy-efficient and continuous processing to reduce water or energy use, maximizing yield to decrease waste, using food waste byproducts, and using food packaging technologies to maximize shelf life.

### Equity

Equity characteristics of SNPFs were also considered in 2 aspects—manufacturing and ingredient sourcing considerations, which include sustainable operations on-farm and throughout the supply chain, as well as attributes related to product formulation and promotion. We agreed that the indicators to include in a definition should focus on living wages and income—both on the manufacturing side (“manufactured and distributed by workers that are paid at least a living wage”) and throughout the supply chain (“grown and harvested by farmers that earn at least a living income and farmworkers that are paid at least a living wage”). There is significant momentum and interest in living wage and income from governments and businesses, but overall, this is still a very nascent area in terms of measurement (which needs to be localized to different regions) and implementation. A safe working environment, freedom of association, and freedom from child labor, forced labor, discrimination, and harassment were also discussed as important considerations in evaluating whether packaged foods were made by companies committed to ensuring fair and healthy working conditions across the supply chain. It was noted that overall, supply chain equity is extremely complex, and although there is alignment on the key issues to address, there are many challenges in assessment.

Regarding the product/promotion domain, we agreed that the highest priority indicators of SNPFs were affordability, desirable taste, texture and appearance, and convenience—easy for consumers to prepare and eat. In terms of affordability, focusing on product purchase price alone can be too simplistic. Consumers are used to paying between 7% and 27% of their income on food, making cost an important consideration for packaged food (38). However, the group felt that rather than include purchase price as the key economic indicator of SNPFs it would be more impactful for the food industry to advocate for broader socioeconomic change, including putting emphasis on paying at least a living wage throughout the supply chain, while acknowledging the importance of innovation to create affordable, accessible SNFPs that meet the needs across population and income groups.

Convenience is an important benefit that packaged foods can provide, and the group suggested that convenience should also include equity considerations—making it possible for working people and caretakers to provide nutritious, sustainable food for themselves and their families. Packaged foods can help make healthy, sustainable diets achievable by helping save time and reduce stress associated with food preparation, meal planning, and procurement, and by making sustainable eating accessible to all skill levels. It was also noted that products should clearly convey product attributes with accessible and easily understood marketing and labeling, which can be further informed by consumer research.

## Application of Guiding Principles to Snacks

Based on our deliberations, we created a set of guiding principles to evaluate and identify SNPFs, as shown in Figure 2, which includes more detailed nutrition criteria for snacks as a specific category of SNPFs. We propose snacks as an initial category given their prevalence and high rate of consumption among packaged foods. There will be distinctions in nutrition guidance based on the purpose of the food (e.g., meal compared with snack). The nutrition criteria exempt performance foods to acknowledge that they are formulated for a specific occasion (e.g., endurance exercise) and thus require different nutrition (39). The principles and criteria proposed here are not intended to be final but are likely to evolve over time as measurement tools advance and our understanding of dietary and sustainability science continues to improve.

## Making Healthy, Sustainable Diets Scalable and Achievable: Potential Paths Forward

We conclude with steps needed to realize the benefits of SNPFs in making healthy, sustainable diets achievable and accessible. It is urgent to accelerate efforts across academia, health professionals, governments, and the food industry, given the scale of food systems transformation needed to address climate change, nutrition insecurity, and inequities. Prioritized actions include: inform and advance evidence-based dietary guidance, definitions, and policies; catalyze broader industry adoption of foods for healthy, sustainable diets; educate professionals and consumers (including messages targeted at those most at risk) on how to identify these foods; and pursue research to refine SNPF definitions and quantify benefits.

Governing documents like the Dietary Guidelines for Americans (DGA) do not include sustainability considerations, despite significant stakeholder efforts and attempts within the 2015 DGA Committee Report (40). The DGA also do not provide recommendations that distinguish among packaged foods designed with nutrition and sustainability in mind. A commentary (41) concludes that nutritionists and dietitians must educate consumers and help inform food policies, guidelines, and labeling that articulate needed changes—potentially discovering that consumers find concerns about environmental impact more motivating than human health when considering dietary change. Success will require considering packaged foods through a broader lens to achieve equitable access and broader adoption of practical healthy and sustainable diets.

Greater public understanding is needed on eating sustainably. Another commentary (42) states that sustainable foods are perceived as expensive, marginalizing acceptance, and that helping consumers identify and select foods that are affordable and convenient, but still nutrient dense and sustainably sourced, would be more effective than discouraging favorite foods. We agree and believe enabling and incentivizing changes in packaged foods (>50% of US diets) to improve impacts will be more effective than trying to encourage more dramatic dietary shifts. An online UK survey (43) found consumers are engaged with some aspects of sustainable diets but remain resistant to others. Several studies highlight the benefits of educating young people on diets (44–47). Thus filling the “public understanding gap” should include a strong youth component.

A commentary (48) highlights the large knowledge gap on the environmental impacts of packaged foods and beverages. Some research has focused on steps needed to make healthy, sustainable diets effective and accessible at scale; but progress has been limited. Although additional research will drive understanding across all packaged foods, our suggested framework for defining SNPFs can serve as a model as science advances. It will help accelerate adoption of healthier, more sustainable eating patterns by providing actionable guidance on how to select packaged foods that consider human, societal, and environmental health.

## Conclusion

There is a growing global consensus around the urgency to improve dietary patterns and how we produce our foods. Until now, the positive role that packaged foods can play has been ignored, and this could be greatly facilitated through further consideration of their nutritional, environmental, and equity impacts in the nutrition policy dialogue. Our workshop deliberations resulted in establishing a framework of guiding principles for defining SNPFs, and we determined that such foods could help make healthy, sustainable diets achievable and accessible, and provide actionable guidance for health professionals and consumers to identify and select these foods. Although ongoing research is essential to further build out and substantiate the definition of SNPFs, adoption and implementation of the proposed framework would play a meaningful role in helping achieve urgent public and environmental health goals.

## Notes

The workshop referenced in this article was sponsored by Clif Bar & Company.

Author disclosures: EAD, AD, MWH, JH, and KAM all received honoraria from Clif Bar & Company for their participation in the meeting summarized here. DIG received funding from Clif Bar & Company for his role as convening lead author on this manuscript. EAD received funding from Potatoes USA for 2 webinars on UPFs. AD is the developer of the Nutrient Rich Food (NRF) index, a nutrient profiling model, and has received grants, contracts, and honoraria from entities, both public and private, with an interest in nutrient density of individual foods, meals, and snacks, and the total diet. MWH is a consultant to Clif Bar & Company and Principal, Flying Goat Associates, LLC.

Abbreviations used: DGA, Dietary Guidelines for Americans; GHG, greenhouse gas; SNPF, sustainable, nutritious packaged food; UPF, ultraprocessed food.
